# 

**Published:** 1888-06

**Authors:** 


### Medical News and Miscellany.

Marriages. Dr. C. M. Rosser, of Waxahachie, was married May 15, to Miss Eva Belknap, of that city, and Dr. L. M. Barnes, of Grandview, to Miss Winburne, in Hill county, May 19, also Dr. Walker to Miss Welsh, in Paris, Texas, May 11.

### GENERAL INDEX—VOL. III.

ORIGINAL CONMUNICATIONS.

Acute Pulmonary Inflammations in Children.............. 320
A Country Doctor's first Case of Surgery............... 42
An enemy of the Bovus attacks Man...................... 25
A Rare Case of Dislocation............................. 85
An Easy Method in Hernia............................... 80
A Novel Theory in Regard to an additional Function of the Liver.......................................... 318
An ugly Enterocele; operation.......................... 40
Antipyrine as a Hæmostatic............................. 369
Acute Dysentery, Treatment of.......................... 369
Abortion, Treatment of................................. 417
Abscess, Perinephritic................................. 420
Amnesic Aphasia, Congestive............................ 171
Cocaine in Urethral Surgery............................ 253
Cocaine in Venereal Surgery............................ 77
Cocaine in Cautery for Venereal Warts.................. 10
Cocaine, Was it the.................................... 520
Case of Quadruplets, A................................. 10
Conjunctivetis; chronic granular....................... 367
Congestion of Brain, followed by Amnesic Aphasia...... 171
Case of Extra Uterine Gestation A...................... 206
Cervical Dilatation in Dysmenehœa...................... 275
Circulation in the Kidney.............................. 460
Chronic Suppuration of Middle Ear...................... 425
Conduct of Medical Societies, The...................... 47
Dysentery, Treatment of................................ 338
Dysentery, Its Causes and Treatment.................... 421
Dysentery, Treatment of Acute.......................... 362
Dengue, Treatment of................................... 309
Errors of Refraction. Eye Disease due to............... 218
Etiology of Infectious Diseases........................ 266
Etiology and Treatment of Talipes...................... 369
Ear-Pain and its Treatment............................. 172
Eye Diseases and other Reflexes........................ 218
Fish Bones in Peritonieal Cavity....................... 216

Fever, Urethan in.................................... 15
Gastrotomy ; Fish Bones in Peritoneal Cavity............ 216
Granular Conjunctivitis............................... 367
Hysteria, A Phenomenal Case of........................ 6
Hemorrhage from the Mouth in a Baby.................. 38
Hot Water in Traumatic Tetanus....................... 75
Heredity, Influence of, etc.............................. 464
Hadra's Operation for Cystocle......................... 82
Hernia, 60 cases of Strangulated........................ 80
Hernia, Strangulated Inguinal, in Inmate of the Asylum.. 133
Hæmaturia Miasmatica................................. 216
Inguinal Hernia, etc....................................... 80
Is the World Satisfied with the Medical Profession?...... 508
Imperforate Hymen, a case of.......................... 1
Little Things in Surgery................................ 376
Live Maggots in Naso-Pharynx.......................... 46
Management and Treatment of the Insane in Texas....... 409
Notes from Obstetric Practice........................... 3
Notes on Electrical Treatment........................... 12
Notes on the Washington Congress..................... 122
Original Observations and Methods in the Practice of Medicine.............................................. 163
Otitis Media, Failure in Treatment..................... 425
Occlusion of the Anus, Congenital...................... 524
Peritonitis, Pelvic ......................................... 503
Prevalent affection much Overlooked................... 575
Peri-Nephritic Abcess................................... 420
Pulmonary Embolisin, Was it?.......................... 136
Pleurodynia............................................... 313
Quadruplets, a case of.................................. 10
Removal of a Shot from the Eye........................ 311
Stomatitis, A Hemorrhage from the Mouth in a Baby..... 35
Septic Fever............................................... 257
Treatment of Internal Hemorrhoids......................———
Traumatic Tetanus ; a case of cured by Hot Water...... 75
Typhlitis Stercoralis, a case of........................ 462
The Conduct of Medical Societies....................... 47
Trachoma—By Dr. F. E. Yoakum, M. D................. 367

CULLINGS FROM CONTEMPORARIES.

........................ 141, 198, 304, 343, 392, 395, 443, 396, 501

CORRESPONDENCE—FOREIGN.

Letter from our own Special Correspondent.........185, 232, 279

CORRESPONDENCE—DOMESTIC.

Asthma, a New Remedy for.............................. 182
A Lusus Naturæ .......................................... 382

A Newsy Letter.................................. 470
Claims for Services at Inquests........................ 183
Chicago, Letter from................................ 275
"Cholera in New York" criticised...................... 325
College, the New Medical, in San Antonio.............. 470
Electricity, value of, as Remedial Agent................ 139
International Med. Congress, letter from Washington on the 89
Letter from Dr. F. T. Paine.......................... 139
" " Dr. L. J. Graham........................ 182
" " Dr. J. E. Mayfield....................... 183
" " Dr. C. H. Hughes ........................ 230
" " Dr. F. E. Yoakum........................ 275
" " Dr. J. L. Felder.......................... 276
" " Dr. A. S. Wolff.......................... 325
" " Dr. J. T. Hutchins on.................... 377
" " Dr. R. P. Talley.......................... 378
" " Dr. E. P. Vellum......................... 380
" " Dr. S. A. Stone.......................... 383
" " Dr. E. N. Mendenhall.................... 382
" " Dr. R. T. Knox........................... 382
" " Dr. B. F. Kingsley....................... 523
" " Dr. H. S. Robertson..................... 584
" " San Antonio.............................. 470
" " Chicago.................................. 275
Malarial Germ, a letter on the ........................ 276
Physicians' Mutual Benefit Associat'n, proposed changes in. 380,383
Remedy, A New, in Asthma............................. 182
Return of Menses in Old Age .......................... 382
Taxation Without Representation ...................... 378
The Loco Plant ...................................... 523
Value of Electricity as a Remedial Agent ............... 139
Veratrum, In Pneumonia ............................... 377

SOCIETY NOTES.

Austin District Med. Soc..............85, 180, 223, 339, 427, 484
Alabama Surgical and Gynecological Ass'n ............ 108
American Public Health Ass'n ......................... 136
American Medical Ass'n, Officers for '88 ............... 487
Annual Meeting West Texas Medical Society ............ 222
Constitution and By-Laws P. M. B. A.................. 389
Cherokee Medical Society.............................. 487
East Texas Medical Society................222, 386, 289, 137, 16
East Line Medical Society ........................289, 222
Lone Star Medical Society ........................387, 486
Ninth International Medical Congress ................ 87, 89
North Texas Medical Society........................... 289
Proceedings Texas State Medical Ass'n.................. 473

Programme Galveston Meeting .......................... 426
Physicians Mutual Benefit Ass'n. 285, 138, 289, 290, 385, 387, 389, 485
Railroad Rates to the I. M. C.......................... 54
Southern Surgical and Gynæcological Ass'n.............. 179
Transactions Texas State Medical Ass'n ................ 84, 55
Texas State Medical Ass'n..................55, 84, 253, 288, 88
Terrell Medical Society..............................134, 340
Travis County Medical Society ....................... 16, 288
ValVerde Medical Society............................ 391
Williamson County Medical Society..................... 288

EDITORIAL.

A Rape on Science.................................... 19
An Unjust Slight..................................... 108
A Roaring Farce...................................... 113
A Scientific Achievement............................. 192
Asylum, State Lunatic The........................... 56, 91
Bury the Hatchet..................................... 437
Biographical.....................................175, 492
Biography of Dr. T. D. Wooten........................ 175
Biography of Dr. J. F. Y. Paine...................... 492
Bibliography / Book Notices } See Separate Heading..................
Cholera in New York, The............................. 146
County Medical Societies of Texas ................... 244
Coming Meeting at Galveston, The..................... 354
Dr. Talley's Letter on Taxation without Representation... 404
Death of Dr. Starley................................. 293
Death of Dr. Gray.................................... 146
Dr. Paine's Discoveries.............................. 194
Dr. Walker's Paper on Hernia......................... 152
Interstate Medical Representation.................... 489
Journalistic Mavericks............................... 108
Lehn & Fink.......................................... 201
Management and Treatment of the Insane in Texas....... 441
Microbes and Microbe Killers......................... 403
Medical College, The Texas........................... 17
Physicians' Mutual Benefit Association Meeting in Galveston.................................... 356
State Health Officer Rutherford...................... 158
Squibbian Inconsistency.............................. 196
Shall the T. S. M. A. have a Permanent Abode?.......... 239
Still at It.......................................... 94
State Medical Library, The Establishment of a.......... 239
Special Committee on Surgery......................... 195
Taxation Without Representation...................... 291

The Galveston Meeting T. S. M. A. .......... 489
The Effort to Secure a Room in the Capitol .......... 489
The Texas Special Committee on Surgery .......... 195
The Vexed Question of Gledetschine .......... 189
Texas Delegates to the Ninth I. M. Congress .......... 20
The State Lunatic Asylum Trouble .......... 91
The Texas Lunatic Asylum and its Management .......... 56

BIBLIOGRAPHY.

Anæmia (by Henry) .......... 105
Athosis, a Satire .......... 297
Annals of Surgery .......... 454
Chart of the Muscular System .......... 71
Companion to U. S. Dispensary .......... 72
Drug Eruptions .......... 104
Diseases of the Hair and Scalp .......... 296
Diseases of the Female Mammary Gland .......... 297
Diseases of the Urethra and Bladder .......... 298
Diseases of the Heart and Lungs .......... 451
Diseases of the Skin; Shoemaker .......... 451
Elementary Microscopic Technology; James .......... 106
Efficacy of Coca Erythroxylon .......... 454
Gonorrhœa and Spermatorrhœa; on the Pathology and Ttreatmant of .......... 299
Health and Home Library .......... 298
Handbook of Treatment .......... 300
Handbook of Medical Sciences, Reference .......... —
Index Catalogue S. G. O. .......... 163
Joseph Jones on Fevers .......... 66
Lesions of the Pelvic Floor .......... 465
Medical World's Visiting List .......... 297
Manual of Physical Diagnosis in Thoracic Disease .......... 299
Manual of Medical Jurisprudence .......... 453
Obstetrics, Practical Treaties on .......... 71
Obstetric, Synopsis .......... 453
Oxygen in Therapeutics .......... 105
Practical Urine Testing .......... 163
Physicians Visiting List .......... 163
Physicians Dose Book .......... 105
Prescription, the .......... 452
Poisoning, What to Do, in case of .......... 70, 297
Quiz. Compend—Obstetrics .......... 72
Renal Disease, a Practical Treatice on .......... 72
Report of Special Committee on Surgery, Texas State Med. Association .......... 70
Text Book in Surgery. Wyeth .......... 67

Transactions Texas State Medical Association........... 95
The Three Ethical Codes.............................. 452
Unwise Laws, Blair................................... 103
University Magazine, the............................. 495
Vick's Floral Guide.................................. 299
Wearing of the Gray.................................. 453

NECROLOGICAL.

Bell, Dr. John....................................... 407
Davis, Dr. J. J...................................... 407
Gunn, Dr. Moses...................................... 203
Gray, Dr. J. A....................................... 146
Glover, Dr. William.................................. 448
Hardin, Dr. W. G..................................... 407
Kilpatrick, Dr. A. R................................. 159
Morton, Dr. John H................................... 156
Nichols, Dr. H. H.................................... 407
Ogle, Dr. S. A....................................... 407
Patillo, Dr. T. M.................................... 407
Starley, Dr. S. F.................................... 293
Wilson, Dr. W. H..................................... 86

BIOGRAPHICAL.

Paine, Dr. J. F. Y................................... 492
Wooten, Dr. T. D..................................... 175

MEDICAL NEWS AND MISCELLANY.

.................22, 60, 107, 153, 197, 205, 300, 358, 405, 496, 447

ADVERTISERS' NOTES.

.................28, 73, 115, 500, 164, 205, 250, 305, 359, 408, 453

